# Tryptophan Breakdown in Patients with HCV Infection is Influenced by IL28B Polymorphism

**DOI:** 10.3390/ph8020337

**Published:** 2015-06-18

**Authors:** Heinz Zoller, Annina Jenal, Albert F. Staettermayer, Sebastian Schroecksnadel, Peter Ferenci, Dietmar Fuchs

**Affiliations:** 1 Department of Internal Medicine, Biocenter, Innsbruck Medical University, Innsbruck 6020, Austria; 2 Division of Biological Chemistry, Biocenter, Innsbruck Medical University, Innsbruck 6020, Austria; 3 Department of Medicine III, Medical University of Vienna, Vienna 1090, Austria

**Keywords:** IL28B polymorphism, tryptophan breakdown, indoleamine 2,3-dioxygenase, kynurenine to tryptophan ratio, neopterin

## Abstract

Until recently, the standard treatment of chronic hepatitis C virus (HCV) infection was a combination therapy with PEG-IFN-α plus ribavirin. Previous studies have proven that several markers predict the outcome of such therapy, e.g., pretreatment plasma levels of interferon inducible protein IP-10, HCV RNA and IL28B-related single nucleotide polymorphisms (SNP). Altered activity of tryptophan metabolizing enzyme indoleamine 2,3-dioxygenase (IDO) has been also shown in patients suffering from HCV infection. In this study, we investigated whether IL28B SNP in patients infected with HCV is related to the tryptophan breakdown rate. Before therapy, serum tryptophan and kynurenine concentrations were determined in 25 patients with established HCV infection and the kynurenine to tryptophan ratio (KYN/TRP) was calculated as an estimate of the tryptophan breakdown rate. In parallel, neopterin and nitrite concentrations were determined. A significant difference of serum KYN/TRP existed between the three IL28B polymorphism groups: C/C genotype had the highest and T/T genotype had the lowest KYN/TRP (*p* < 0.05). Likewise, C/C genotype was associated with higher KYN/TRP than non-C/C genotype (*p* = 0.01). There was a smaller difference between the three groups regarding the absolute kynurenine concentrations, the C/C genotype being associated with higher kynurenine concentrations. None of the other comparisons revealed any statistical significance. In conclusion, patients with C/C genotype presented with the highest tryptophan breakdown rate already before antiretroviral therapy with IFN-α/ribavirin. The differences in tryptophan metabolism might relate to HCV clearance and also to side effects of IFN-α therapy.

## 1. Introduction

Until recently, the standard treatment of chronic hepatitis C virus (HCV) infection involved pegylated interferon-α (IFN-α) plus direct antiviral substances with or without ribavirin, and sustained suppression of HCV is feasible in approximately over 90% of patients with all-oral combination [1,2]. A genome-wide screening identified IL28B gene nucleotide polymorphisms to be associated with HCV clearance [3,4,5,6]). Single nucleotide polymorphisms (SNPs) in the 19q13 region, in close proximity to three genes (IL28A, IL28B, and IL-29) encoding cytokines of the IFN-l (*i.e.*, type III IFN) family, predict spontaneous clearance of HCV infection [5,7,8]. IL28B encodes for IFN-lambda-3 (IFN-l-3), which is potentially involved in the pro-inflammatory response. IL28B polymorphism is not only associated with HCV clearance but was also found to relate to adverse effects of IFN-α which limits its clinical use [9].

Multiple genes and biochemical effector pathways mediate the antiviral activity of IFN-α during therapy [10], breakdown of essential amino acid tryptophan may represent one of the key components. The tryptophan-degrading enzyme indoleamine 2,3-dioxygenase (IDO) is strongly induced by pro-inflammatory cytokines [11,12]. Thereby interferon-γ (IFN-γ) is the most potent *in vitro*-stimulus of tryptophan breakdown in human monocyte-derived macrophages [13], whereas in human dendritic cells IFN-α, IFN-β and IFN-γ were found to act equally strong [14].

Within the Th1-type immune response, tryptophan breakdown is devoted to limit availability of the essential amino acid which restricts proliferation of invading pathogens or tumor cells [15]. Activation of IDO has been already demonstrated to possess strong antimicrobial and antiviral activity [16,17], and moreover enhanced tryptophan breakdown as is indicated by an increased kynurenine to tryptophan ratio (KYN/TRP) was already described in patients suffering from chronic HCV infection [18,19]. In parallel to IDO, IFNs stimulate several other biochemical pathways which counteract cell proliferation, among them inducible nitric oxide synthase (iNOS) is of comparable importance as IDO [20] and the IFN-inducible GTP-cyclohydrolase I (GCH-I, EC 3.5.4.16), that gives rise to production of pteridines [21]. Thereby most human cells and cells from other species form 5,6,7,8-tetrahydrobiopterin (BH_4_), cofactor of several monoxygenases including iNOS, whereas high output neopterin production at the expense of BH_4_ is observed in human monocyte-derived macrophages and dendritic cells [22].

In order to investigate whether IL28B polymorphism is related to IDO activity and to the innate immune host response, we determined the rate of tryptophan breakdown in serum of 25 patients with established HCV infection by measurement of tryptophan and kynurenine concentrations and calculated KYN/TRP as an estimate of the tryptophan breakdown rate. In parallel, concentrations of macrophage marker neopterin and nitrite levels were determined.

## 2. Experimental Section

### 2.1. Patients

Twenty-five patients (12 females, 13 males) with confirmed HCV infections were included in this study. They were aged 41.9 ± 12.1 years (mean ± SD; Table 1). Fibrosis stage was assessed by transient elastography using Fibroscan equipped with an M-probe. In patients in whom transient elastography was technically impossible or where the results did not pass quality control (*i.e.*, IQR of 10 measurements > 25% or success rate < 80%), clinical assessment for the presence of cirrhosis was made. These patients are labelled. Patients without clinically detectable cirrhosis were labelled “no cirrhosis”. As indicated treatment response could not be assessed in all patients, because some patients were lost to follow up.

**Table 1:** Demographics, IL28B genotype, HCV load and biomarker concentrations in the study population.

Age (y)	Sex	Fibrosis status	IL28B genotype	HCV genotype	HCV LOG10	Therapy Response *
23.5	Female	No cirrhosis	T/T	3a	5.36	SVR
24.5	Female	No cirrhosis	C/T	3a	5.67	SVR
25.2	Female	F0-1	T/T	1a	5.50	LFU
30.1	Male	F0-1	T/T	3a	5.34	SVR
30.4	Female	F0-1	C/T	1a	5.75	SVR
30.6	Female	F0-1	C/C	1b	7.02	Relapse
31.1	Male	F0-1	C/C	3a	4.64	SVR
32.5	Female	No cirrhosis	C/T	3a	5.82	LFU
34.0	Female	F0-1	C/T	4	3.90	SVR
38.9	Male	F2	T/T	4	5.05	SVR
39.8	Male	F0-1	T/T	1a	6.91	No response
39.9	Female	F0-1	C/T	3a	5.93	SVR
40.7	Male	F3	C/T	3a	4.72	Relapse
42.3	Male	No cirrhosis	C/T	3a	5.62	SVR
45.1	Female	No chirrhosis	C/C	3a	3.43	SVR
45.3	Female	F3	C/T	3a	6.53	SVR
48.0	Male	Chirrhosis Child a	C/C	1b	5.36	SVR
48.2	Male	F0-1	C/T	1a	7.52	Relapse
49.1	Male	F3	C/T	3a	6.34	Relapse
54.0	Female	F0-1	C/C	1a	7.01	Relapse
54.0	Male	F3	C/C	3a	6.61	Relapse
54.8	Male	Chirrhosis Child A	C/T	3	6.48	Relapse
55.0	Female	F3	C/T	1b	6.16	SVR
60.0	Male	Chirrhosis Child A	C/C	2	6.30	SVR
70.1	Female	F2	C/C	1b	4.13	LFU

* LFU = lost through follow-up, SVR = sustained viral response.

The patients were referred to initiate treatment with pegylated IFN-α plus ribavirin, but did not receive this therapy until blood was drawn for this study. Samples were stored at −80 °C until thawed for biological assays. The candidate C/T single-nucleotide polymorphism upstream from IL28B (rs1297860) was genotyped, and the distribution of CD28B polymorphisms was 8 C/C, 12 C/T, 5 T/T.

All participants’ rights were protected, and according to the Helsinki Declaration, informed consent was obtained that a small portion of their blood collected for routine examinations was forwarded for further scientific testing.

### 2.2. Laboratory Variables

HCV load was determined by quantitative polymerase chain reaction using the Roche assay (COBAS AmpliPrep/COBAS TaqMan HCV Test, v2.0, Roche, Basel, Switzerland). Tryptophan and kynurenine concentrations were measured by high performance liquid chromatography as described [23]. After precipitation of protein with trichloroacetic acid, tryptophan was measured by detection of its native fluorescence at 285 nm excitation and 365 nm emission wavelengths. Kynurenine and internal standard l-nitrotyrosine were monitored by UV-absorption at 360 nm wavelength. Standard preparations containing tryptophan, kynurenine and nitrotyrosine in the presence of albumin underwent the whole procedure like serum specimens. To estimate the breakdown rate of tryptophan, the ratio of the concentrations of the enzyme product kynurenine to the substrate tryptophan (KYN/TRP) was calculated [24]. In addition, neopterin concentrations were measured by enzyme-linked immunosorbent assay (BRAHMS GmbH, Hennigsdorf, Germany) [25]. To estimate production of nitric oxide (NO**^.^**), the stable NO**^.^**metabolite nitrite (NO_2_^−^) was determined in the sera by the Griess reaction assay [26].

### 2.3. Statistical Analysis

Statistical comparisons were made using non-parametric tests because some of the data sets did not show normal distribution: Kruskal-Wallis test was used for comparison of several groups, and Wilcoxon paired rank test for comparisons of two groups only. Two-sided Spearman rank correlation was applied to test for associations between variables. *P*-values below 0.05 were considered to indicate significant differences or associations.

## 3. Results

Average ± S.D. HCV load in patients was 5.7 ± 1.0 lg copies/µL. Tryptophan concentrations were 51.4 + 14.6 µmol/L *vs.* 67.4 ± 10.2 µmol/L in healthy controls of similar age distribution (Geisler *et al.* [27]) (*p* < 0.001), and kynurenine concentrations were 2.1 ± 1.0 µmol/L *vs.* 1.78 ± 0.42 µmol/L in healthy controls (*p* < 0.05), resulting in KYN/TRP of 41.6 ± 22.3 µmol/mmol *vs.* 26.7 ± 6.2 µmol/L in healthy controls (*p* < 0.01). Neopterin concentrations were 7.8 ± 8.7 nmol/L and higher than normal (*p* < 0.05) and nitrite levels were 11.9 ± 21.2 µmol/L.

**Figure 1:**
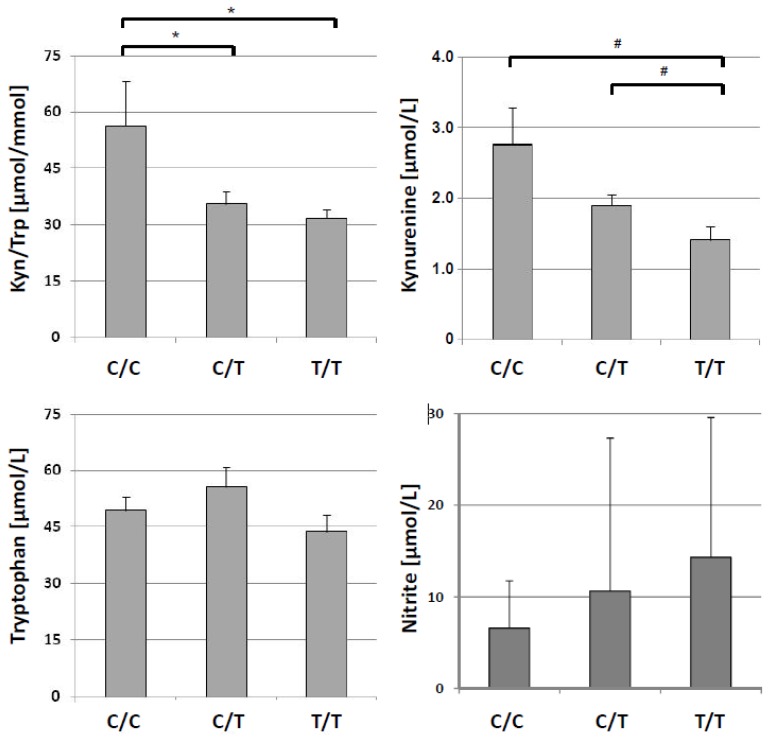
Distribution of concentrations of kynurenine to tryptophan (KYN/TRP, upper left) kynurenine (**upper right**), tryptophan (**lower left**) and nitrite (**lower right**) concentrations in patients with HCV infection split into 3 groups according to IL28B genotypes C/C, C/T and T/T (mean + S.E.M. is shown for each genotype, * *p* < 0.05, ^#^ *p* <0.06).

When comparing different subgroups according to IL28B polymorphisms, kynurenine concentrations were found to significantly differ between the three groups (Χ² = 6.22, *p* <0.05; Table 2). Further analysis revealed that the C/C genotype was associated with higher KYN/TRP (56.4 ± 33.7 µmol/mmol, *n* = 8) than the C/T genotype (35.7 ± 10.9 µmol/mmol, *n* = 12; U = 2.08, *p* <0.05) or the T/T genotype (31.9 ± 4.51 µmol/mmol, *n* = 5; U = 2.20, *p* < 0.05), see Figure 1.

There was no difference between C/T and T/T genotypes regarding KYN/TRP. C/C genotype was associated with higher KYN/TRP than non-C/C genotype (C/C 33.7 ± 9.50 µmol/mmol; U = 2.447, *p* = 0.01). Although the range of KYN/TRP in the 8 IL28B CC patients is wide (individual levels: 25.7, 36.0, 46.3, 47.1, 47.2, 52.8, 59.9, 136 µmol/mmol), 2/8 patients with CC genotype had higher ratios (>37 µmol/mmol) than the highest ratio found in TT. On the other hand, CT genotype was associated with in-between levels, 8/12 patients presenting with KYN/TRP above the threshold level.

There was only a trend of a difference between the three groups for kynurenine concentrations (Χ² = 5.63, *p* = 0.060), C/C genotype presented with higher kynurenine concentrations (2.76 ± 1.48 µmol/L, *n* = 8) than the T/T genotype (1.41 ± 0.44 µmol/L, *n* = 5; U = 2.08, *p* < 0.05), and there was a tendency towards a difference between C/C and C/T genotypes (U = 1.90, *p* < 0.06, Figure 1).

**Figure 2:**
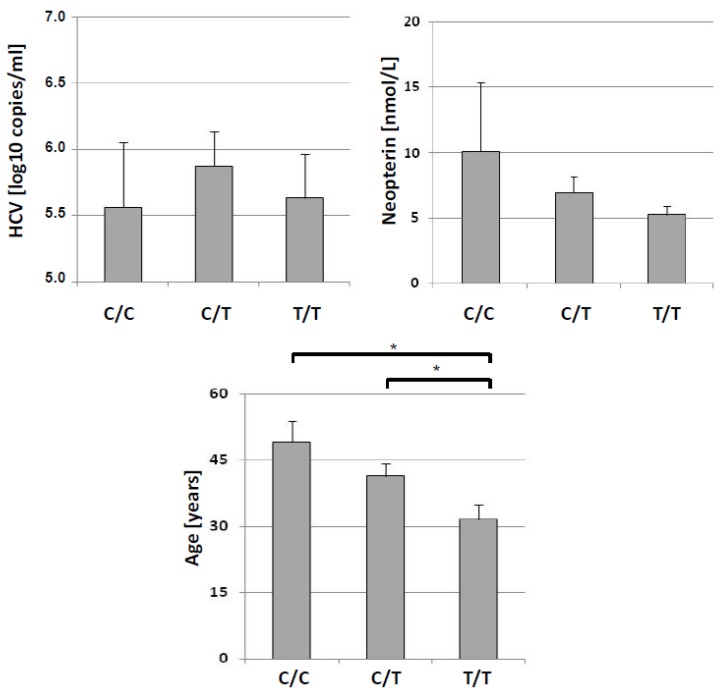
Distribution of HCV load (upper left), neopterin concentrations (upper right) and age in patients (lower) with HCV infection split into three groups according to IL28B genotypes C/C, C/T and T/T (mean + S.E.M. is shown for each genotype, * *p* < 0.05).

Mean neopterin concentrations also showed a tendency towards higher levels in patients with C/C genotype than in patients of the other two groups (Figure 2), but this difference did not reach statistical significance. The opposite trend was observed for nitrite with lower levels in patients with C/C genotype as compared with the other 2 groups (Figure 2), but again failed to reach the level of statistical significance. Likewise, HCV load was not different between any of the genotypes (all *p*-values > 0.200).

Patients with the C/C genotype were older (49.1 ± 13.6 years) than those with C/T (41.4 ± 9.67) or T/T genotype (31.5 ± 7.57; U = 7.044, *p* < 0.05), and also C/C genotype patients were older than the patients with other than C/C genotype (U = 2.446, p = 0.014). Older age was associated with higher kynurenine concentrations (rs = 0.470, *p* < 0.05), but there was neither such association of age with KYN/TRP nor (rs = 0.283, n.s.) with neopterin (rs = 0.065, n.s.).

There was no statistically significant relationship between any of the biomarkers of tryptophan metabolism and HCV load (all *p* > 0.150). By contrast, neopterin concentrations correlated inversely with HCV load (rs = −0.406, *p* < 0.05) without interferon therapy, indicating that higher neopterin concentrations at baseline were associated with SVR independent of IL28B status. There was also a trend towards a correlation with nitrite levels (rs = 0.369, *p* = 0.070). Notably there was no significant correlation between KYN/TRP and neopterin concentrations.

## 4. Discussion

IL28B polymorphism is a major determinant of treatment efficacy with pegylated IFN-α + ribavirin and has also been found to predict treatment response in patients receiving direct antivirals [28]. Not only HCV clearance but also side effects of therapy were observed to be influenced by IL28B genotype, e.g., genotype C seemed to show better treatment response and less side effects of therapy [5]. Existing data indicate that antiviral and neuropsychiatric biochemical pathways are differently influenced in patients according to their genotype. Alterations of tryptophan metabolism could play a key role for both aspects, because on the one hand, cytokine induced tryptophan breakdown represents a key element of the antiviral immune responses and also in immunoregulation [17,29]. On the other hand, tryptophan availability is central for neurotransmission as a precursor molecule of neurotransmitter 5-hysdroxytryptamine (5HT, serotonin) and of neurotoxins like quinolinic acid and of nicotinamide adenine dinucleotides NAD/NADH [30,31,32].

An increased tryptophan breakdown rate has been described in patients with HCV infection and was regarded as a result of activated IDO due to an antiviral immune response [18,19]. IDO activity was further accelerated during IFN-α + ribavirin treatment [33]. In our cohort of untreated patients with HCV infection an accelerated tryptophan breakdown rate was indicated by higher KYN/TRP as compared with healthy controls, results agreeing well with data from the literature [18,19]. However, neither kynurenine nor KYN/TRP and tryptophan concentrations correlated with neopterin levels. This is an unusual observation because in earlier studies in several groups of patients with, e.g., human immunodeficiency virus infection, autoimmune syndromes like rheumatoid arthritis or systemic *lupus erythematosus* or various types of cancer, close positive correlations between KYN/TRP and neopterin production were reported [34]. And such a close relationship between an immune activation marker like neopterin and KYN/TRP can be regarded as an indicator for an activated IDO when a pro-inflammatory stimulus would induce tryptophan breakdown in parallel with neopterin production in macrophages. However, the lack of correlation between neopterin and KYN/TRP in our study cannot confirm this relationship. It may suggest that other cells than macrophages, e.g., hepatocytes are important for IDO activity and/or that the second tryptophan-degrading enzyme, namely hepatic tryptophan 2,3-dioxygenase (TDO), could be involved in the enhancement of KYN/TRP [30]. Both aspects are supported by the fact that HCV infection is strongly affecting liver metabolism and could represent part of an immune evasion strategy of HCV. Moreover, in an earlier study it was found that while neopterin levels in HCV infected patients correlated significantly with endogenous IFN-γ levels, this correlation disappeared under therapeutic administration of IFN-α [35].

IL28B genotype was found to influence kynurenine concentrations and KYN/TRP, C/C genotype being associated with highest levels whereas patients with C/T genotype presented with lower levels, and lowest levels were observed in T/T genotype patients (Table 2). Neopterin concentrations showed some trend towards the same direction (Figure 1), the differences of neopterin levels did not reach the level of statistical significance. Data shows that it is not just the treatment response which is influenced by IL28 polymorphism, already the baseline tryptophan breakdown rate was different between groups. Similar observations were made earlier when concentrations of IFN-γ inducible protein 10 kDa (IP-10 or CXCL10) were compared [36,37]. With this background of a significant association between neopterin and IFN-γ concentrations in HCV infection [35] one would expect that significant alterations of neopterin concentrations existed between patients with different genotypes, but although the mean values of neopterin concentrations seemed to follow the same trend as KYN/TRP, the number of patients studied was obviously too small and the study might be underpowered to detect such a difference statistically.

**Table 2:** HCV load, tryptophan, kynurenine, nitrite and neopterin concentrations as well as the kynurenine to tryptophan ratio (KYN/TRP) in patients with HCV infection grouped according to IL-28B polymorphisms (mean ± S.D. are shown; n.s. = not significant).

IL28B Polymorphism	C/C	C/T	T/T	non-C/C	Χ^2^, *p* *	U, *p* **
	(*n* = 8)	(*n* = 5)	(*n* = 12)	(*n* = 17)		
HCV load [log 10 copies/µL]	5.56 ± 1.38	5.87 ± 0.92	5.63 ± 0.73	5.80 ± 0.85	0.183, n.s.	0.146, n.s.
Age [y]	49.1 ± 13.6	41.4 ± 9.67	31.5 ± 7.57	38.5 ± 10.1	7.044, 0.030	1.806, 0.075
Tryptophan [µmol/L]	49.5 ± 10.5	55.8 ± 17.7	43.8 ± 9.54	52.2 ± 16.4	2.603, n.s.	0.058, n.s.
Kynurenine [µmol/L]	2.76 ± 1.48	1.92 ± 0.55	1.41 ± 0.44	1.77 ± 0.55	5.627, 0.060	1.748, 0.086
Kyn/Trp [µmol/mmol]	56.4 ± 33.7	35.7 ± 10.9	31.9 ± 4.52	33.7 ± 9.50	6.218, <0.05	2.447, 0.014
Nitrite [µmol/L]	6.58 ± 5.16	10.6 ± 16.7	14.32 ± 15.3	8.17 ± 10.1	0.916, n.s.	0.233, n.s.
Neopterin [nmol/L]	10.1 ± 14.8	6.96 ± 4.30	5.28 ± 1.53	6.47 ± 3.73	0.805, n.s.	0.175, n.s.

* *p*-values, comparison of C/C, C/T and T/T groups, Kruskal Wallis test; ** *p*-values, comparison of C/C *vs.* non-C/C groups, Mann Whitney U-test/.

Nitrite concentrations were highest in patients with the T/T genotype, lower in C/T and lowest in C/C genotype (Table 2), but again the differences between groups were not significant. Higher nitrite levels are considered to allow some conclusions about NO**^.^** production rates [38]. The determination of nitrite concentrations is regarded to be superior to nitrate or nitrite+nitrate measurements because nitrates are not only final products of NO**^.^** oxidation via nitrites, but could also be produced from peroxynitrites, formed upon the reaction of NO with oxygen free-radicals like superoxide anion (O_2_^−^). Since it is well known that NO**^.^** production could affect IDO expression and activity [39], one might expect to exist an inverse relationship between KYN/TRP and nitrite concentrations. Indeed the comparison between IL28B genotypes gives the impression that mean values of nitrite behave in a mirror shaped from when compared to KYN/TRP, kynurenine or neopterin concentrations. However, no significant relationship was found in our cross-sectional data set when correlation analysis was performed. Again the small number of patients studied might prevent to detect such a difference by statistics.

In our study, there existed a significant relationship between IL28B polymorphism and tryptophan breakdown expressed as KYN/TRP. Tryptophan breakdown could thus play a role in the distinct IFN-α responses of patients according to the C/C, C/T and T/T genotypes. Especially the observation that C/C genotype was associated with higher KYN/TRP than non-C/C genotype is in good agreement with the observed treatment response in patients according to IL28B polymorphism. Differences in tryptophan metabolism could contribute to the findings regarding HCV clearance, because tryptophan breakdown represents an important antiviral mechanism. Also influence of IL28B polymorphism on side effects of IFN-α therapy could easily relate to a distinct biochemical background which is pre-existing in untreated patients. Earlier studies have proven that IL28B polymorphism is a major determinant of treatment efficacy in patients treated with PEG-IFN-α plus ribavirin [40,41]. Not only HCV clearance but also side effects of therapy were observed to be influenced by IL28B genotype. So the C allele in rs1297860 was associated with both, improved viral clearance with more frequently sustained viral response but also with more somatic complaints, like loss of energy, worsened sleep and change in appetite [36,37,41,42]. Tryptophan availability is crucial for neurotransmission as a precursor molecule of serotonin and of neurotoxins like quinolinic acid and of nicotinamide adenine dinucleotides NAD [30,31,32].

Also in patients with HIV and HCV co-infection, low pre-treatment levels of IP-10 are associated with significantly higher sustained viral response rates upon IFN-α therapy than patients with high IP-10 levels. Surprisingly in our study, kynurenine levels and KYN/TRP were highest in patients with the C/C genotype which is usually an indicator of superior treatment response. So the question arises whether IDO activity as is indicated by higher KYN/TRP is representative for the immunosuppressive consequences of the enzyme rather than its relationship to IFN production. Like IDO, IP-10 has an antiviral and immunosuppressive function by activating the innate immune system. This may also explain the fact that patients with lower levels of IP-10 have a higher first-phase decline in HCV RNA, so these are good virological responders and high pre-treatment IP-10 were associated with non-response [40,41].

IFNs are well known to activate the kynurenine pathway of tryptophan metabolism. The results of this study suggest a previously unknown association between response to antiviral treatment and activity of tryptophan metabolism. Literature data pointed to an inverse correlation between antiviral response and concentrations of proteins, production of which is encoded by interferon-stimulated genes [41]. In the absence of treatment response data we can only speculate that IFN-induced expression of genes is of greater relevance in the control of HCV than previously thought. Also the induction of neopterin and ß2-miocroglobulin was found previously to relate to outcome of therapy with IFN-α2b [35]. Those patients with lower baseline levels of the biomarkers were those who responded better to IFN-α therapy when a greater increase of neopterin and B2M concentrations can be achieved. Moreover, the rate of response to antiviral therapy was reported to be higher among HCV patients with lower pretreatment neopterin levels [42]. Neopterin predicted rate of response even after controlling for HCV genotype status, one the strongest predictors of response to treatment. In our study, there was a statistically significant inverse relationship between HCV load and neopterin concentrations before IFN therapy, indicating that in the natural history of HCV infection higher baseline neopterin concentrations were associated with SVR independent of IL28B status. Data might correspond to an endogenous immune response against HCV which is to some part able to control virus production rates.

Former studies revealed that patients’ age influenced the viral clearance upon therapy [43,44]. Our patients with the C/C genotype were older than patients with the T/T genotype, and according to literature there exists a relationship between older age and higher tryptophan breakdown rates and usually also between kynurenine and KYN/TRP with neopterin concentrations that is most probably due to increasing inflammation with older age [45]. However, in our patients there were no such correlations between increased tryptophan breakdown and age or neopterin concentrations. Still older age could contribute to the association of kynurenine levels and KYN/TRP with IL28-2B genotypes. Data may indicate that in patients with C/C genotype HCV infection progresses to a slower degree than in patients with other genotypes thus seeking the physician and hospital later.

### Limitations

The number of patients investigated in this study is still low and so the results of the study need to be confirmed in independent and larger study cohorts. The predictive value of tryptophan metabolic changes regarding treatment response still needs to be elaborated.

## 5. Conclusions

There exists a significant relationship between IL28B polymorphism and kynurenine production due to tryptophan breakdown. Tryptophan breakdown could thus play a role in the distinct IFN-α responses of patients according to the C/C, C/T and T/T genotypes. Differences in tryptophan metabolism could relate to HCV clearance, because tryptophan breakdown represents an important antiviral mechanism. Also influence of IL28B polymorphism on neuropsychiatric side effects of IFN-α therapy could easily relate to a distinct biochemical background, which is pre-existing in untreated patients. Moreover, cirrhosis/stage, age, HCV genotype, viral load and gender are important and could all influence the values reported (see characteristics of patients in Table 1), but could not be adequately controlled for. However, we analyzed baseline values only and any conclusion about a relationship between tryptophan breakdown and treatment response cannot be drawn. Such a relationship still needs to be demonstrated. One might speculate that spontaneous clearance or fibrosis progression might relate to the immune mechanisms which were found to exist already before the treatment of patients with IFN. This is especially of relevance when meanwhile IL28B genotype has lost prognostic impact in nearly all phase 3 trials.

In sum the results of our study can only be regarded as preliminary and the number of patients still needs to be extended to be able to prove an association between the IL28B polymorphism and the tryptophan breakdown rate and the possible involvement of IDO.
